# P-2059. Association of Race and Ethnicity With Antibiotic Resistance

**DOI:** 10.1093/ofid/ofaf695.2223

**Published:** 2026-01-11

**Authors:** Deepali Tailor, Matthew Sims

**Affiliations:** Oakland University William Beaumont School of Medicine, San Luis Obispo, CA; William Beaumont University Hospital, Corewell Health, Royal Oak, Michigan

## Abstract

**Background:**

Understanding factors which lead to antibiotic resistance is critical to improving clinical practice. There is limited prior research on the association of race and ethnicity and antibiotic resistance and how such social determinants of health contribute to the development of antibiotic resistance. Here we report the results of a retrospective analysis of antibiotic resistance in Gram-negative pathogens from patients of a large healthcare system in Southeast Michigan and the association of antibiotic resistance to patient race and ethnicity.

**Methods:**

For the year 2019, we identified 29,836 unique adult patients from Corewell Health facilities in Southeast Michigan with cultures that grew Gram-negative bacteria. The antibiograms were evaluated and overall resistance rates to gentamicin, tobramycin, cefepime, piperacillin-tazobactam, meropenem, and ciprofloxacin were stratified by patient race and ethnicity as recorded in the electronic medical record. Race was categorized as White, Black, Asian, Native American/Alaska Native, Pacific Islander, or Other. Ethnicity was categorized as Hispanic/Latino, Arab/Middle Eastern, Not Hispanic/Latino, and Other. Patients with missing race or ethnicity were excluded from analysis.

**Results:**

Black race and Arab/Middle Eastern ethnicity had higher rates of antibiotic resistance to the 6 antibiotics analyzed compared to other races and ethnicities. Asian race had lower rates of resistance to all antibiotics studied except for ciprofloxacin which was higher than all other races. Despite having lower overall resistance to the beta lactams, Asian race had the highest percentage of extended spectrum beta lactamase producing bacteria.

**Conclusion:**

These findings show an association between race, ethnicity, and antibiotic resistance in Gram-negative pathogens. This provides evidence that social determinants of health contribute to antibiotic resistance. We suspect these associations relate to customs, diet, and economic factors. Future research should focus on whether such associations are seen in other geographic regions and understanding the underlying mechanisms driving these disparities in order to help mitigate the development of antibiotic resistance in diverse populations.

**Disclosures:**

Matthew Sims, MD, PhD, Affinity Biosensors: Grant/Research Support|Applied Biocode: Advisor/Consultant|Applied Biocode: Grant/Research Support|Astra Zeneca: Grant/Research Support|Biotest AG: Grant/Research Support|Inflarx: Advisor/Consultant|Janssen: Grant/Research Support|Leonard-Meron Biosciences: Grant/Research Support|Locus Biosciences: Grant/Research Support|Pfizer: Grant/Research Support|PhAST Corp: Grant/Research Support|Prenosis: Advisor/Consultant|Prenosis: Grant/Research Support|QIAGEN: Grant/Research Support|Seres: Advisor/Consultant|Seres: Grant/Research Support|Vedanta Biosciences: Grant/Research Support

